# Chromosome-level genome assembly of the Asian aspen *Populus davidiana* Dode

**DOI:** 10.1038/s41597-023-02350-5

**Published:** 2023-07-06

**Authors:** Eun-Kyung Bae, Min-Jeong Kang, Seung-Jae Lee, Eung-Jun Park, Ki-Tae Kim

**Affiliations:** 1 Department of Forest Bioresources, National Institute of Forest Science, Suwon, 16631 Republic of Korea; 2 Division of Biotechnology, College of Life Sciences and Biotechnology, Korea University, Seoul, 02841 Republic of Korea; 3 Department of Agricultural Life Science, Sunchon National University, Suncheon, 57922 Republic of Korea

**Keywords:** Plant ecology, Forest ecology, Comparative genomics

## Abstract

The genome of *Populus davidiana*, a keystone aspen species, has been sequenced to improve our understanding of the evolutionary and functional genomics of the *Populus* genus. The Hi-C scaffolding genome assembly resulted in a 408.1 Mb genome with 19 pseudochromosomes. The BUSCO assessment revealed that 98.3% of the genome matched the embryophytes dataset. A total of 31,862 protein-coding sequences were predicted, of which 31,619 were functionally annotated. The assembled genome was composed of 44.9% transposable elements. These findings provide new knowledge about the characteristics of the *P. davidiana* genome and will facilitate comparative genomics and evolutionary research on the genus *Populus*.

## Background & Summary

Forest trees in natural populations are excellent materials for accessing the genomic architecture of evolutionary adaptation because they are mostly undomesticated and ecologically important across a wide variety of habitats and harbor abundant genetic and phenotypic variation^1,2^. The genus *Populus* (~30 species), including aspens, poplars, and cottonwoods, has a global geographic distribution throughout the Northern Hemisphere. Aspens and poplars are pioneer species with the fastest growth rates observed in temperate tree species, partly due to their characteristic heterophyllous growth^3^. Moreover, poplars show significant genetic variation among sections, species, individuals, and populations within the genus due to the pollen and seed airborne dispersal mechanism and their obligate outcrossing nature (dioecious)^4^. These traits, enhanced in interspecific hybrids, make an important contribution to meeting the global need for paper, biofuel, timber, bioremediation, and animal feed^4^. Due to its small genome size (less than 500 Mb), adequacy for genetic transformation, ease of propagation, and rapid growth, *Populus* has been established as an efficient model system for studies of forest tree species^5,6^.

The advance of *Populus* as a model system for woody perennial plants has been mainly caused by the rapid development of genomic and molecular biology resources from the *Tacamahaca* section of the *Populus* genus. This includes completion of the reference genome sequence of *Populus trichocarpa* (black cottonwood)^7^, *P. euphratica* (desert poplar)^8^, *P. pruinose* (sister of desert poplar)^9^. While draft genome sequences for two aspen species, *P. tremula* (European aspen)^3^ and *P. tremuloides* (American aspen)^3^, are available, their genome assemblies using a hybrid approach that merged 454 and Illumina short read sequencing were highly fragmented (No. of scaffolds = 216,318 for *P. tremula* and 164,504 for *P. tremuloides*)^3^. *P. davidiana* is another sibling species belonging to the same section of the genus *Populus* (section *Populus*) along with the two aspen species^10,11^. Previous phylogenetic studies revealed that *P. tremuloides* diverged earlier than the other aspen species, *P. tremula* and *P. davidiana*, due to the break-up of the Bering Land bridge^12,13^. After that, the uplift of the Qinghai-Tibetan Plateau and associated climate fluctuations may have driven the divergence between *P. davidiana* and *P. tremula*^12^. In addition, hybridization can readily occur in these aspen species, and resulting artificial hybrids exhibit heterosis for many wood characteristics^14^, suggesting that the speciation process has not been completed among the three aspen species^13^. Therefore, it is crucial to understand how different evolutionary forces have shaped the genomic landscape of differentiation along the forest tree speciation continuum. The high-quality reference genome resources from the *Populus* section, such as *P. davidiana*, will shed light on the phenomenon.

Here, we present a high-quality chromosome-level *de novo* genome assembly for the Asian aspen species *Populus davidiana* Dode. This new assembly will greatly improve genome completeness and contiguity over the previous aspen genomes. Furthermore, access to the *P. davidiana* genomic data set will facilitate research on the speciation continuum of *Populus* species and accelerate the breeding speed of forest trees by leveraging unexplored adaptive gene repositories.

## Methods

### Sample preparation and DNA sequencing

**Fig. 1:**
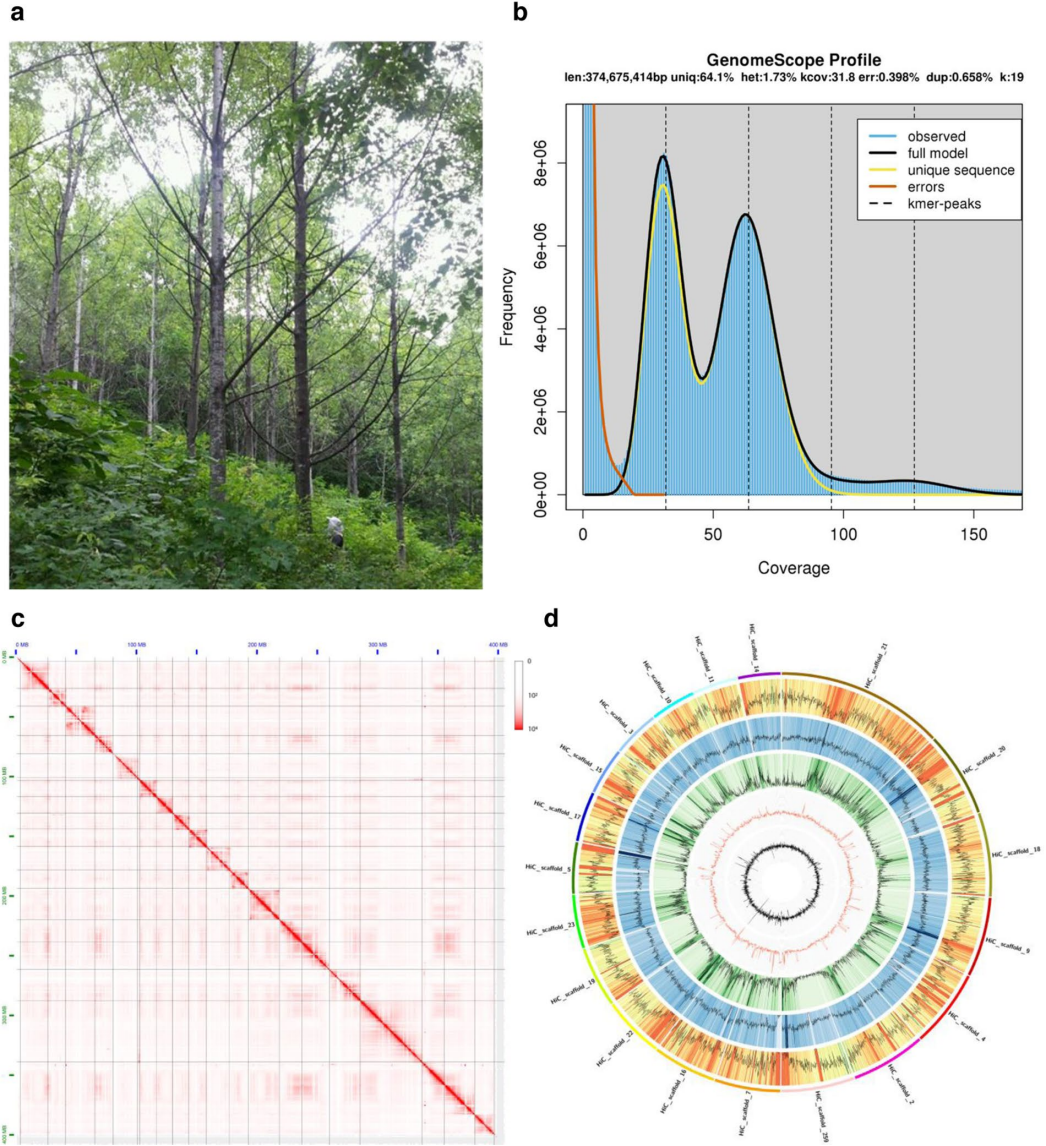
The sample and genome of *P. davidiana*. (**a**) Photograph of a 27-year-old female *P. davidiana* tree located in Youngju (36°49′N 128°37′E; 575-m altitude) in Gyeongsangbuk-do Province, Republic of Korea. (**b**) Genome characteristics of *P. davidiana* using GenomeScope. (**c**) Hi-C interaction heatmap and overview of the *P. davidiana* genome. The 19 assembled scaffolds are ordered by length. The x- and y-axes provide the mapping positions for the first and second reads in each read pair, respectively, grouped into bins. The color of each square indicates the number of read pairs within that bin. Grey lines have been added to indicate the borders between scaffolds. (**d**) The features are arranged in the order of gene density, repeat density, LTR/Gypsy, GC contents, and GC skew from outside to inside in 1 Mb intervals across the 19 chromosomes.

Fresh leaves of *P. davidiana* were collected from a 27-year-old female tree (Odae 19) in a clonal seedling located in Youngju (36°49′N 128°37′E; 575-m altitude) in Gyeongsangbuk-do Province, Republic of Korea (Fig. 1a). High-molecular-weight genomic DNA (gDNA) was isolated from the sample using the modified cetyltrimethylammonium bromide (CTAB) method^15^. The quality and quantity of the extracted DNA were then determined using a 2100 Bioanalyzer (Agilent Technologies, Santa Clara, CA, USA). The genomic survey was performed using an Illumina paired-ended DNA library (550 bp insert), following the Illumina TruSeq DNA PCR-Free Library Prep protocol (Illumina, San Diego, CA, USA). The library was checked by Agilent 2100 Bioanalyzer High Sensitivity Kit and then sequenced on the Illumina NovaSeq6000 platform using a 150-bp paired-end strategy.

For HiFi sequencing, one 8 M SMRTbell DNA libraries were constructed using the following steps, according to the PacBio HiFi library construction protocol: (i) gDNA target size shearing using Megaruptor 3 (Diagenode); (ii) DNA damage repair; (iii) blunt-end ligation with hairpin adapters from the SMRTbell Express Template Prep Kit v2 (101-685-400, PacBio, Menlo Park, CA, USA); (iv) size-selection using the BluePippin Size Selection System (Sage Science, Beverly, MA, USA); and (v) binding to polymerase using the Sequel II Binding Kit v2.2 (Pacific Biosciences, Menlo Park, CA, USA). Subsequently, HiFi sequencing was performed on a PacBio Sequel II platform with the Sequel II Sequencing Kit v2.

A Dovetail Hi-C library was constructed and sequenced with the Illumina NovaSeq6000 platform, following published protocols^16^. Hi-C fragment libraries were prepared using the ‘Proximo Hi-C protocol’ with *Dpn*II digestion, and the resulting libraries were sequenced using a 150-bp paired-end strategy.

### Genome assembly

The original Illumina paired-ended sequencing produced 35.7 Gb and 17.8 Gb of clean data after filtering out low-quality reads containing poly-*N* and adapter sequences using FASTP v.0.12.6 (set to default parameters)^17^ (Supplementary Table 1). The trimmed sequencing reads were used to calculate the percentage of heterozygosity in the genome. First, Jellyfish v.2.2.10^18^ was used to compute a histogram of 19 k-mer frequencies (count -F 2 -m 19 -C -s 10 G). Then, heterozygosity was calculated using the GenomeScope v.2.0 online platform^19^. The platform predicted genome size of 374.7 Mb, with a heterozygosity of 1.73% (Fig. 1b). In addition, the long-read sequencing of the *P. davidiana* genome obtained 862,684 PacBio HiFi reads (11,636.6 Mb) representing a sequencing depth of 31.1X (Supplementary Table 1).

For *de novo* genome assembly, the FALCON-Unzip assembler was used with length cutoff parameters (length cutoff = 13 kb, length cutoff pr = 10 kb) and filtered subreads from SMRT Link v.5.0.0 (minimum subread length = 50 bp)^20^. To improve the accuracy of assembly, the Arrow algorithm was implemented using the unaligned BAM files as raw data to polish the FALCON-Unzip assembler. The *de novo* assembly resulted in a genome size of 498.7 Mb with a contig N50 of 2.3 Mb (Table 1). In addition, Purge Haplotigs was used to remove duplicated haplotypes as haplotigs from the whole-genome sequencing data^21^. The high, mid, and low cutoff read depth parameters were set to 170, 55, and 5 to remove haplotigs (default parameter). Consequently, the genome assembly contained 407.9 Mb in 484 polished contigs with an N50 of 2.74 Mb, and the GC content of the genome was 34.87% (Table 1).

**Table 1:** Assembly statistics of the *P. davidiana* genome.

	FALCON-Unzip	Purge Haplotigs	HiRise
Number of contigs (scaffolds)	935	484	259
Total size of contigs (scaffolds)	498,655,568	407,852,175	408,135,175
Longest contig (scaffold)	11,725,180	11,725,180	51,652,470
Number of contigs (scaffold) > 1 M nt	131	122	22
Number of contigs (scaffold) > 10 M nt	2	2	19
N50 contig (scaffold) length	2,317,531	2,742,334	20,279,470
L50 contig (scaffold) count	61	43	8
GC contents (%)	35.43	34.89	34.87

The Hi-C fragment library sequencing produced 44.42 Gb (118.5X coverage) of clean data (Supplementary Table 1). The Dovetail Hi-C reads and the draft assembly were used as input data for HiRise (default parameter), a pipeline designed for scaffolding genome assemblies by utilizing proximity ligation data^22^. SNAP read mapper was used to align Hi-C library sequences to the draft input assembly^23^. Error correction was performed using Pilon^24^ with the short-read data, and organelle genomes were filtered out using BLAST v.2.4.0^25^ (-max_target_seqs. 1 -evalue 0.001). A total of 259 assembled contigs were anchored onto 19 pseudochromosomes ranging from 13.1 to 51.7 Mb in length, containing 96.4% of the genome sequences (Fig. 1c; Supplementary Table 2). The final genome had N50 of 20.3 Mb, the highest among the sequenced *Populus* species (Supplementary Table 3). Finally, Benchmarking Universal Single-Copy Orthologs (BUSCO) v.4.1.2 was used to assess the completeness of the genome assembly (Table 2)^26^.

**Table 2:** Statistics for genome assessment using BUSCO (embryophyta).

	# of BUSCOs	% of BUSCOs
Complete	1,587	98.3
Complete and single-copy	1,348	83.5
Complete and duplicated	239	14.8
Fragmented	7	0.4
Missing	20	1.3

### Transcriptome sequencing

Three types of tissue samples, including leaf, stem, and root, were collected from *P. davidiana*. The samples were immediately stored in liquid nitrogen at −80 °C until RNA extraction. Total RNAs were extracted from each sample using TRIzol reagent (Invitrogen, Waltham, MA, USA), and their purity and integrity were checked using the Bioanalyzer 2100 system (Agilent Technologies, Santa Clara, CA, USA). RNA sequencing libraries were prepared according to the manufacturer’s instructions (Illumina Truseq stranded mRNA library prep kit). mRNA was purified and fragmented from total RNA using poly-T oligo-attached magnetic beads with two rounds of purification. Cleaved RNA fragments primed with random hexamers were reverse transcribed into first-strand cDNA using reverse transcriptase, random primers, and dUTP in place of dTTP. The products were purified and enriched with PCR to create the final strand-specific cDNA library. After QPCR using SYBR Green PCR Master Mix (Applied Biosystems), we combined libraries that index tagged in equimolar amounts in the pool. Finally, RNA sequencing was performed using an Illumina NovaSeq6000 system following the provided protocols for 2 × 100 sequencing. The RNA sequencing produced 15.9 Gb of raw read data (Supplementary Table 1).

### Protein-coding gene annotation

The *P. davidiana* genome was annotated using *ab initio* gene prediction, custom repeat library protocols, homology search, and full-length transcript evidence. The MAKER v.2.31.8 pipeline was used for genome annotation, with three rounds of reiterative training^27^. Initially, the pipeline was run in ‘est2genome’ mode based on the transcriptome assembly which was generated by Trinity v2.8.5 from the RNA-seq data^28^. Additionally, *ab initio* gene prediction was performed using Augustus^29^ and SNAP^30^ (snaphmm = A.thaliana.hmm augustus_species = <BUSCO retraining model>). Finally, Exonerate v2.4.0 was implemented to polish MAKER alignments with evidence for protein-coding genes obtained from the genomes of three *Populus* species: *P. trichocarpa*, *P. alba*, and *P. euophratica*^31^. The best-supported gene models were selected based on the Annotation Edit Distance (AED) quality metric developed by the Sequence Ontology project^32^. The final genome assembly consisted of 19 pesudochromosomes and contained 31,862 protein-coding genes with an AED score less than 0.5 (Table 3). The final gene set had an average of 5.8 exons per gene, with a total length of 38.9 Mb and an average length of 1,219.6 bp.

**Table 3:** Statistics for *P. davidiana* genome annotation.

Features	# of Features	Total Length of Features (bp)	Average Length of Features (bp)	Density (#/Mb)
Gene	31,862	114,415,598	3,590.97	78.1
CDS	31,882	38,882,028	1,219.56	78.1
Exon	185,916	50,074,248	269.34	45.6
Intron	154,034	64,365,548	417.87	37.7
3′ UTR	22,496	3,677,853	163.49	5.5
5′ UTR	25,630	7,514,367	293.19	6.3

Although *P. davidiana* had the highest N50 value among the sequenced *Populus* genomes, it had the lowest number of predicted genes (Supplementary Table 4). On the other hand, *P. tremula* had the second-best N50 value and the most genes among the poplar species, with 37,184 genes^33^. However, the gene density of *P. davidiana* genome was 78.1 genes per Mb (Fig. 1d; Table 3), which was not the lowest among the sequenced *Populus* species. *P. euphratuca* and *P. tremuloides* had the lowest and the highest gene density, respectively, with 69.82 and 96.3 genes per Mb (Supplementary Table 4). The highest density feature of *P. euphratuca* may be due to the relatively low genome quality^8^.

**Fig. 2:**
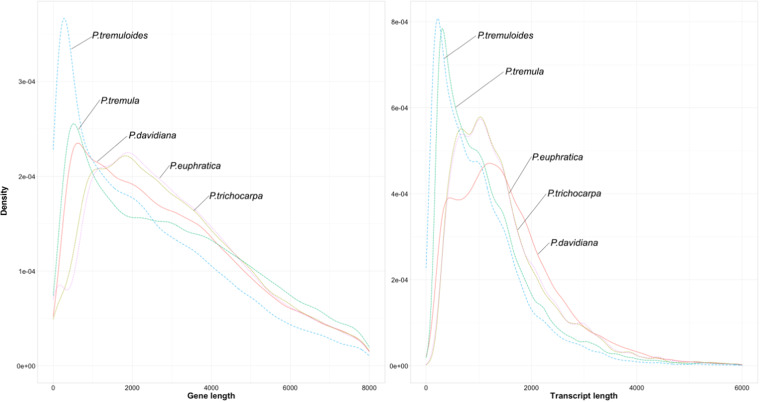
The gene and transcript length distribution of *P. davidiana* and the other four *Populus* species (*P. trichocarpa*, *P. euphratica*, *P. tremula*, and *P. tremuloides*).

The density of genes and transcripts was analyzed based on their length distribution among different *Populus* species (Fig. 2). *P. tremuloides*, *P. tremula* and *P. davidiana* had many genes with short lengths (<1.0 kb). In contrast, genes with a length of around 1.9 kb were most abundant in *P. euphratica* and *P. trichocarpa*. The transcript length distribution was similar to the gene length distribution pattern, except for *P. davidiana*. It had the lowest frequency of both short- and long-length transcripts, indicating a relatively short length of CDS compared to the other *Populus* species (Supplementary Table 4).

Functional annotation was performed using the predicted genes as queries. BLAST v.2.4.0 was run with a maximum e-value cutoff of 1e-5 against the National Center for Biotechnology Information (NCBI) UniProtKB/Swiss-Prot database^25^. In addition, InterProScan v.5.44.79^34^ and BLAST2GO-based gene ontology (GO) analysis^35^ were used to annotate the predicted proteins. The Kyoto Encyclopedia of Genes and Genomes (KEGG) database was also consulted for KEGG functional annotations in BLAST2GO^35,36^. Most CDSs (31,619 proteins, 99.2%) were annotated by the UniProt database (Supplementary Table 5). InterProScan annotated functions of 30,463 proteins (95.55%), and the other tools, including Pfam, GO, and KEGG, annotated 11,983 (37.6%), 15,966 (50.1%), and 3,039 (9.6%) proteins, respectively (Supplementary Table 5). The Third-level GO term analysis of the predicted proteome revelaed that proteins involved in cellular metabolic processes, intracellular anatomical structure, and organic cyclic compound binding were the most abundant in *P. davidiana* genome.

### Repeat and non-coding RNA annotation

A *de novo* repeat library was created with the default parameters of RepeatModeler v.1.0.3, which includes RepeatScout v.1.0.5^37^ and RECON v.1.08^38^. Tandem Repeats Finder v.4.09^39^ was used to predict repetitive sequences and classify information for each repeat, including low-complexity repeats, satellites, and simple repeats (default parameter). An LTR library was constructed with LTR_retriever^40^, using combined raw LTR data from LTR_FINDER and LTRharvest to identify highly accurate long terminal repeat retrotransposons (LTR-RTs)^41,42^. Finally, RepeatMasker v.4.0.9^43^ was used to identify repetitive elements in the *de novo* repeat library and Kimura distances were calculated for all transposable element (TE) copies from each family found in the library to estimate the age of TEs^44^ (-lib -no_is).

**Fig. 3:**
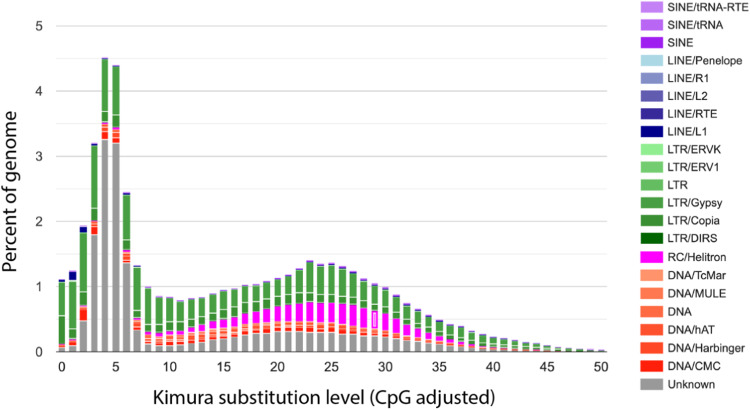
Kimura distance-based copy divergence analysis of TEs in *P. davidiana* genomes. The graphs represent genome coverage (y-axis) for each type of TEs (DNA transposons, SINE, LINE, and LTR retrotransposons) in Kimura substitution level (CpG adjusted) illustrated on the x-axis (K-value from 0 to 50). The color chart indicates the repeat types.

Retrotransposable elements, which are known to be the dominant form of repeats in angiosperm genomes^45^, constituted 47.9% (195.5 Mb) of the *P. davidiana* genome (Fig. 3; Table 4). This is higher than those of other *Populus* sections, such as *P. tremula* (43.1%) and *P. tremuloides* (39.2%). Class I (retrotransposons) and Class II (DNA transposons) TEs accounted for 23.1% and 5.87% of the genome, respectively. Like other sequenced *Populus* genomes, LTR retrotransposons, mainly Gypsy-type and Copia-type LTRs, were predominant (22.68%), and with other DNA elements (DNAs) accounted for 4.86% of the genome. Of the repetitive elements, 10.91% could not be classified into any known families, indicating that *P. davidiana*, and perhaps the poplar family in general, may contain many novel repetitive or transposable elements.

**Table 4:** Sequence percentage (%) of annotated TEs of the *P. davidiana* and four other Populus species.

		*P. davidiana*	*P. tremula*	*P. tremuloides*	*P. euphratica*	*P. trichocarpa*
DNA transposon	DNA	4.86	5.60	5.74	4.25	6.98
	LINE	1.01	1.06	0.62	0.86	1.01
Retro-transposon	SINE	0.43	0.50	0.52	0.39	0.53
	LTR	22.68	21.38	13.61	29.29	22.43
	Gypsy	13.43	12.71	8.80	24.29	15.79
	Copia	5.17	5.60	4.86	4.27	5.18
Other	Unknown	10.91	8.21	10.87	6.72	8.43
	Total	47.90	43.10	39.21	46.87	46.89

Notes: LINE, long interspersed nuclear element; SINE, short interspersed nuclear element; LTR, long terminal repeat.

Other non-coding RNAs and putative tRNA genes were identified using the Barrnap v0.9 (https://vicbioinformatics.com/software.barrnap.shtml) and tRNAscan-SE v2.0.5^46^, respectively. Lastly, the number of rRNAs and tRNAs predicted from *P. davidiana* genome were 2,879 and 683, respectively.

## Data Records

The *P. davidiana* genome project has been deposited in the NCBI database under BioProject accession PRJNA833418. The genome assembly data have been deposited at GenBank under the WGS accession JAMQGN000000000^47^. The sequencing reads are available at the Sequence Read Archive (SRA) under accessions from SRR24038974 to SRR24038979 (SRP430397)^48^. In addition, the genome, predicted transcripts and proteins, structural and functional annotation files (gff files), and results from repeat analysis had been deposited in FigShare^49^.

## Technical Validation

The primary contigs and haplotigs of the draft FALCON-Unzip and the Purge Haplotigs-processed assemblies were evaluated using the BUSCO pipeline based on the embryophyta_odb9 database (Supplementary Table 6). Although the total number of BUSCOs was similar for both assemblies, the Purge Haplotigs haploid assembly had 12.5% more single-copy BUSCOs and 12.8% fewer duplicated BUSCOs than the draft FALCON-Unzip assembly. BUSCO assessment of the final genome assembly found that 1,587 (98.3%) of the 1,614 highly conserved orthologs were present as complete genes. This included 1,348 (83.5%) single-copy BUSCOs and 239 (14.8%) duplicated BUSCOs (Supplementary Table 6).

## Supplementary information

Supplementary Tables

## Data Availability

We followed the developers’ instructions for the bioinformatics tools used in this study. The software and code used are publicly accessible, with the version and parameters used specified in the Methods section. No custom code was used during the compilation of the dataset.
