# Supplementary material for: Chromosome-level genome assembly of the Asian aspen Populus davidiana Dode
Source: Sci Data. 2023 Jul 6;10:431. doi: 10.1038/s41597-023-02350-5 (PMC10326025; doi:10.1038/s41597-023-02350-5)
Supplement: Supplementary file 1 — Supplementary Tables [file 41597_2023_2350_MOESM1_ESM.docx]

**Supplementary Table 1.** Sequencing data used *P. davidiana* genome assembly

|  | **Platform** | **Insert size** | **# of Bases** | **Sequencing coverage (x)** |
| --- | --- | --- | --- | --- |
| DNA | PacBio Sequel (HiFi) | 15Kb | 11,635,637,837 | 31.05 |
|  | Illumina Novaseq6000 | 550bp | 35,658,610,740 | 95.17 |
|  | Hi-C | - | 44,416,577,520 | 118.54 |
| RNA | Illumina Novaseq6000 | 100bp | 15,942,376,310 | 42.55 |

**Supplementary Table 2.** Lengths of *P. davidiana* genome assembly

| **Scaffolds** | **Length(bp)** | **% of Total Bases** |
| --- | --- | --- |
| HiC_scaffold_21 | 51,652,470 | 12.66 |
| HiC_scaffold_20 | 26,359,530 | 6.46 |
| HiC_scaffold_18 | 25,826,000 | 6.33 |
| HiC_scaffold_9 | 24,566,559 | 6.02 |
| HiC_scaffold_4 | 24,224,153 | 5.94 |
| HiC_scaffold_2 | 23,019,158 | 5.64 |
| HiC_scaffold_259 | 22,600,641 | 5.54 |
| HiC_scaffold_7 | 20,279,470 | 4.97 |
| HiC_scaffold_16 | 19,706,000 | 4.83 |
| HiC_scaffold_22 | 18,937,280 | 4.64 |
| HiC_scaffold_19 | 18,777,520 | 4.60 |
| HiC_scaffold_23 | 16,517,028 | 4.05 |
| HiC_scaffold_5 | 15,825,054 | 3.88 |
| HiC_scaffold_17 | 15,427,000 | 3.78 |
| HiC_scaffold_15 | 14,510,000 | 3.56 |
| HiC_scaffold_3 | 14,438,015 | 3.54 |
| HiC_scaffold_11 | 13,771,453 | 3.37 |
| HiC_scaffold_14 | 13,093,470 | 3.21 |
| Total | 393,446,490 | 96.40 |

**Supplementary Table 3.** Genome assemblies used in this study

| **Common name** | **Species** | **Size (Mb)** | **# of scaffolds (Chromosome)** | **Scaffold N50** | **Reference** |
| --- | --- | --- | --- | --- | --- |
| Desert poplar | *Populus euphratica* | 496.0 | 9,615 | 482,055 | Ma et al., 2013 |
| Desert poplar | *Populus pruinosa* | 479.3 | 78,960 | 698,525- | Yang et al., 2017 |
| Black cottonwood | *Populus trichocarpa* | 434.3 | 1,694(19) | 2,158,892 | Tuskan et al., 2006 |
| European aspen | *Populus tremula* | 408.7 | 1,601(19) | 16,928,776 | Schiffhaler et al., 2019 |
| Asian aspen | *Populus davidiana* | 408.1 | 259(19) | 20,279,470 | This study |
| American aspen | *Populus tremuloides* | 377.5 | 164,504 | 15,222 | Lin et al., 2018 |
| Purple willow | *Salix purpurea* | 329.3 | 348(20) | 14,688,223 | Goodstein et al., 2012 |
| Thale cress | *Arabidopsis thaliana* | 119.7 | 7(5) | 23,459,830 | The Arabidopsis Genome Initiative. 2000 |

Ma et al. 2013. Genomic insights into salt adaptation in a desert poplar. *Nat. Commun.* 4(2797): 1-8. doi: 10.1038/ncomms3797

Yang et al. 2017. The draft genome sequence of a desert tree *Populus pruinose*. *GigaScience.* 6(9): 1-7. doi: 10.1093/gigascience/gix075

Tuskan et al. 2006. The genome of black cottonwood, *Populus trichocarpa* (Torr. & Gray). *Science*, 313(5793): 1596-604. doi: 10.1126/science.1128691

Schiffthaler et al. 2019. An improved genome assembly of the European aspen *Populus tremula*. *bioRxiv*, 805614. doi: 10.1101/805614

Lin et al. 2018. Functional and evolutionary genomic inferences in *Populus* through genome and population sequencing of American and European aspen. *Proc. Natl. Acad. Sci. U.S.A.* 115(46): E10970-E10978. doi: 10.1073/pnas.1801437115

Goodstein et al. 2012. Phytozome: a comparative platform for green plant genomics, *Nucleic Acids* *Res.* 40(D1): D1178-D1186. doi: 10.1093/nar/gkr944

The Arabidopsis Genome Initiative. 2000. Analysis of the genome sequence of the flowering plant *Arabidopsis thaliana*. *Nature* 408: 796–815. doi: 10.1038/3504869

**Supplementary Table 4.** Statistics of annotations for four species

| **Species/Features** | **Number of genes** | **Total length of genes (bp)** | **Average length of genes (bp)** | **Percentage of total length of genes (%)** | **Number of CDSs** | **Total length of CDSs (bp)** | **Average length of CDSs (bp)** | **Percentage of total length of CDSs (%)** | **Density (#/Mb)** |
| --- | --- | --- | --- | --- | --- | --- | --- | --- | --- |
| *P.trichocarpa* | 35,416 | 138,860,196 | 3,921 | 31.97 | 51,717 | 77,289,113 | 1,494 | 17.80 | 81.55 |
| *P.euphratica* | 34,620 | 142,931,509 | 4,129 | 28.82 | 49,760 | 73,776,669 | 1,483 | 14.88 | 69.82 |
| *P.tremula* | 37,184 | 153,661,350 | 4,132 | 37.59 | 73,765 | 87,948,975 | 1,192 | 21.51 | 90.95 |
| *P. tremuloides* | 36,361 | 99,237,577 | 2,729 | 26.29 | 35,694 | 36,681,670 | 1,028 | 9.72 | 96.30 |
| *P.davidiana* | 31,862 | 114,415,598 | 3,591 | 28.03 | 31,882 | 38,882,028 | 1,220 | 9.53 | 78.07 |

**Supplementary Table 5.** Search for database search with the *P. davidiana* annotations

| **Database** | **# of mRNAs** | **% via Total mRNAs** |
| --- | --- | --- |
| Uni-prot/swiss-prot | 31,619 | 99.18 |
| InterProScan | 30,463 | 95.55 |
| Pfam | 11,983 | 37.59 |
| GO | 15,966 | 50.08 |
| KEGG | 3,039 | 9.53 |

**Supplementary Table 6.** Statistics for genome assessment using BUSCO (embryophyta)

|  | **FACON-Unzip** | | **Purge haplotigs** | **Hi-C** |
| --- | --- | --- | --- | --- |
| Complete | | 1,591 (98.6%) | 1,587 (98.3%) | 1,587 (98.3%) |
| Complete and single-copy | | 1,146 (71.0%) | 1,348 (83.5%) | 1,348 (83.5%) |
| Complete and duplicated | | 445 (27.6%) | 239 (14.8%) | 239 (14.8%) |
| Fragmented | | 4 (0.2%) | 7 (0.4%) | 7 (0.4%) |
| Missing | | 19 (1.2%) | 20 (1.3%) | 20 (1.3%) |
